# Assessment of Subclinical Doxorubicin-induced Cardiotoxicity in a Rat Model by Speckle-Tracking Imaging

**DOI:** 10.5935/abc.20170097

**Published:** 2017-08

**Authors:** Yu Kang, Wei Wang, Hang Zhao, Zhiqing Qiao, Xuedong Shen, Ben He

**Affiliations:** Renji Hospital, School of medicine, Shanghai Jiaotong University, Shanghai, China

**Keywords:** Cardiotoxicins, Oxidative Stress, Doxorubicin, Echocardiography, Doppler, Troponin I

## Abstract

**Backgrounds:**

Despite their clear therapeutic benefits, anthracycline-induced cardiotoxicity is a major concern limiting the ability to reduce morbidity and mortality associated with cancers. The early identification of anthracycline-induced cardiotoxicity is of vital importance to assess the cardiac risk against the potential cancer treatment.

**Objective:**

To investigate whether speckle-tracking analysis can provide a sensitive and accurate measurement when detecting doxorubicin-induced left ventricular injury.

**Methods:**

Wistar rats were divided into 4 groups with 8 rats each, given doxorubicin intraperitoneally at weekly intervals for up to 4 weeks. Group 1: 2.5 mg/kg/week; group 2: 3 mg/kg/week; group 3: 3.5mg/kg/week; group 4: 4mg/kg/week. An additional 5 rats were used as controls. Echocardiographic images were obtained at baseline and 1 week after the last dose of treatment. Radial (Srad) and circumferential (Scirc) strains, radial (SRrad) and circumferential (SRcirc) strain rates were analyzed. After the experiment, cardiac troponin I (cTnI) was analyzed and the heart samples were histologically evaluated.

**Results:**

After doxorubicin exposure, LVEF was significantly reduced in group 4 (p = 0.006), but remained stable in the other groups. However, after treatment, Srads were reduced in groups 2, 3 and 4 (p all < 0.05). The decrease in Srads was correlated with cTnI (rho = -0.736, p = 0.000) and cardiomyopathy scores (rho = -0.797, p = 0.000).

**Conclusion:**

Radial strain could provide a sensitive and noninvasive index in early detection of doxorubicin-induced myocardial injury. The changes in radial strain had a significant correlation with myocardial lesions and serum cardiac troponin I levels, indicating that this parameter could accurately evaluate cardiotoxicity severity.

## Introduction

Cardiotoxicity, which may result from cardiac oxidative stress, is the main limiting factor of the anticancer therapy using anthracycline.^1^ Noninvasive techniques for the identification of patients who are at high risk of developing anthracycline-induced cardiomyopathy are critically important for the prevention and management of this complication.

Currently, two-dimensional speckle-tracking imaging (STI), based on tracking local image details from frame to frame throughout the cardiac cycle, has been reported as a simple and accurate method for the assessment of left ventricular mechanics.^2-5^ It has been applied for early detection of myocardial injury in ischemic heart disease or various cardiomyopathies in both humans and experimental animals, allowing more accurate measurements of regional myocardial systolic performance.^6-10^

The purpose of this study was to determine, by means of an experimental rat model using doxorubicin, whether STI could provide a more sensitive and accurate measurement in detecting left ventricular injury.

## Methods

### Animal treatment

This protocol was approved by the Animal Care and Use Committee of the Shanghai Jiaotong University and was in compliance with the "Guide for the Care and Use of Laboratory Animals" published by the National Academy Press. Thirty-seven adult male Wistar rats, weighing 250.4 ± 4.3 g, were housed at constant temperature, with freely available food and water. The sample size calculation was performed based on the following assumptions: after anthracycline exposure, the difference in strain values was 20% between groups, the standard deviation within the group was 10%, power was 0.80, and the significance level was 0.05. As a result, we calculated a required sample size of 8 rats in each treatment group. Rats were randomized into 4 groups with 8 rats each, based on the process published by Martin RA et al.,^11^ given doxorubicin intraperitoneally at weekly intervals for up to 4 weeks. Group 1: 2.5 mg/kg/week, total dose 10 mg/kg; group 2: 3 mg/kg/week, total dose 12 mg/kg; group 3: 3.5mg/kg/week, total dose 14 mg/kg; group 4: 4 mg/kg/week, total dose 16 mg/kg. An additional 5 rats were used as controls, which received 1 mL of 0.9% saline solution intraperitoneally.

### Echocardiography protocol

Images were obtained at baseline and 1 week after the last dose of anthracycline treatment. Rats were anesthetized by an intraperitoneal injection of 10% chloral hydrate at a dose of 0.3 ml/Kg. The rats were put in left lateral decubitus position and scanned using a commercially available echo-scanner, the Vivid ultrasound cardiovascular system (GE Healthcare Inc., Horten, Norway.), using a 10S (11.5MHz) phased array pediatric transducer and a cardiac application with high temporal and spatial resolution. The transmission frequency was 10MHz, the depth 2.5 cm, and the frame rate was 225 frames per second. The standard two-dimensional (2D) short-axis images acquired at the papillary muscle level were digitally stored for further off-line analysis. Left ventricular dimensions were measured using M-mode echocardiography through the short-axis view of the mid-papillary level and left ventricular ejection fraction (LVEF) was calculated using the Teicholz method.

EchoPAC 11.0 (GE Healthcare In,. Norway) was used for radial strain (Srad), circumferential strain (Scirc), radial strain rate (SRrad), and circumferential strain rate (SRcirc) analysis. This 2D-strain program tracked the movement of strong reflectors that were observed in the B-mode images, frame by frame, after segmenting the ventricular silhouette into six segments. The endocardial border was marked, while the outer border was adjusted to fit the epicardial contour. The software automatically tracked and computed strain and strain rate in radial and circumferential directions throughout the cardiac cycle. Peak systolic Srad, Scirc, SRrad and SRcirc were obtained from 6 segments of the papillary muscle levels. Data of at least three distinct cardiac cycles were averaged.

### Histological study

One week after the end of the doxorubicin administration, the animals were euthanized with an overdose of chloral hydrate. Blood samples were collected for determination of serum levels of cardiac troponin I (cTnI). Left ventricles at the level of papillary muscles were fixed in phosphate-buffered 10% formalin, embedded in paraffin, and sectioned at a thickness of 5µm. These sections were stained with hematoxylin and eosin. The frequency and severity of myocardial lesions induced by doxorubicin were assessed semiquantitatively by light microscopic examination. The changes were graded based on the number of myocytes showing myofibrillar loss and cytoplasmic vacuolization (score from 0 to 3 according to Billingham.^12^) Animals that died spontaneously during the study also underwent necropsy, but they were not included in the data analysis.

### Serum levels of cTnI

Blood samples were centrifuged and the serum samples were frozen at -80°C until analyzed. Serum concentrations were determined by immunoassay (Denley Dragon Wellscan MK 3, Thermo, Finland). The cardiomyopathy scores were calculated by an expert and cTnI levels were recorded by a technician, who were blinded to the experimental process and echocardiographic data.

### Statistical analysis

Continuous variables close to a normal distribution were expressed as the mean ± standard deviation. Non-normal, skewed data were expressed as medians and boundaries of interquartile ranges. One sample K-S test was used to determine the normality of data. Differences in echocardiographic data before and after treatment, and between each group were determined using repeated measure ANOVA analysis. Values of cTnI levels and cardiomyopathy scores between each group were analyzed by Kruskal Wallis test. Spearman analysis was used in determining the correlation between strain values, cTnI and cardiomyopathy scores. Data were analyzed using the SPSS software, version 16.0 (SPSS, Inc, Chicago, IL, USA). A value of p < 0.05 was considered significant.

## Results

### General toxicity and gross anatomic changes

One of the rats from group 3 died after the third dose of doxorubicin. No terminal blood sample of this animal was available, and thus it was excluded from the study. At the necropsy, excessive amounts of pericardial and peritoneal fluids were observed in 4 of 8 animals from group 2, in 6 of 7 animals from group 3 and all animals from group 4. Excess fluid was also observed in the animal that died spontaneously. Accumulation of fluid was not found in the animals from group 1 and animals that received saline solution.

### Systolic functions

There was no significant difference in LVEF at baseline between the groups. After doxorubicin exposure, LVEF reduced from 85.50 ± 1.06% to 82.50 ± 1.85% (p = 0.006) in animals given 16mg/kg doxorubicin. However, LVEF in the other animals receiving lower doses of doxorubicin showed no statistical difference before and after treatment (Table 1).

**Table 1:** LVEF and speckle-tracking indices in doxorubicin-treated and control rats

	Control group			Group 1			Group 2			Group 3			Group 4		
	Baseline	After	P value	Baseline	After	P value	Baseline	After	P value	Baseline	After	P value	Baseline	After	P value
LVEF (%)	85.4 ± 0.9	84.8 ± 2.9	0.591	86.2 ± 1.9	86.4 ± 2.3	0.890	84.6 ± 3.3	84.3 ± 2.9	0.714	83.9 ± 2.4	83.3 ± 2.3	0.220	85.5 ± 1.2	82.5 ± 1.8*	0.006
Srad (%)	52.2 ± 3.6	52.6 ± 3.1	0.730	51.2 ± 6.8	49.4 ± 5.2	0.061	52.1 ± 5.6	43.2 ± 5.7*	0.000	52.5 ± 5.1	38.6 ± 4.8*	0.000	52.3 ± 7.3	34.6 ± 7.4*	0.000
Scirc (%)	–17.2 ± 3.1	–18.2 ± 4.6	0.551	–16.1 ± 2.0	–17.0 ± 2.2	0.113	–17.2 ± 2.4	–16.7 ± 2.4	0.578	–17.0 ± 2.7	–17.9 ± 2.0	0.634	–17.4 ± 2.1	–14.1 ± 1.8*	0.004
SRrad (sec^-1^)	5.7 ± 1.1	5.5 ± 1.2	0.821	5.9 ± 0.8	6.1 ± 1.2	0.617	6.0 ± 0.9	6.0 ± 1.1	0.983	5.6 ± 1.1	5.6 ± 1.1	0.987	5.6 ± 1.0	5.5 ± 1.1	0.786
SRcirc (sec^-1^)	4.5 ± 0.7	4.9 ± 0.5	0.137	4.5 ± 1.2	4.7 ± 0.9	0.556	4.3 ± 1.0	4.3 ± 0.8	0.571	4.6 ± 0.9	4.3 ± 0.5	0.409	4.7 ± 0.7	4.5 ± 0.7	0.179

LVEF: left ventricular ejection fraction; Scirc: circumferential strain; Srad: radial strain; SRcirc: circumferential strain rate; SRrad: radial strain rate.

* p < 0.05 compared with that of baseline.

### Strain analysis

**Figure 1:**
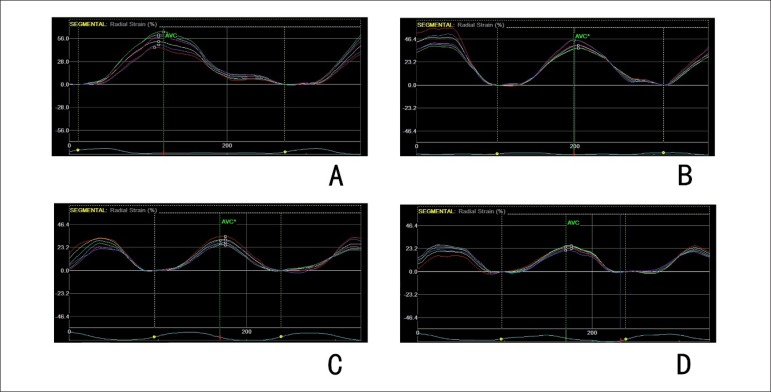
Radial strain curves obtained at the short-axis view of rats after doxorubicin treatment. A: rat from group 1, radial strain = 55.23%; B: rat from group 2, radial strain = 41.63%; C: rat from group 3, radial strain = 29.71%; D: rat from group 4, radial strain = 24.95%.

Data on the strain and strain rate values in animals in the various treatment groups were summarized in Table 1. Baseline characteristics of doxorubicin-treated animals were similar to those of controls. Radial strains reduced after treatment in animals of group 2, group 3, and group 4 (from 52.1 ± 5.6% to 43.2 ± 5.7%, 52.5 ± 5.1% to 38.6 ± 4.8% and 52.3 ± 7.3% to 34.6 ± 7.4% respectively, all p values < 0.05). Circumferential strain reduced from -17.4 ± 2.1% to -14.1 ± 1.8% in group 4 after treatment (p = 0.004). The reduction of radial strains induced by doxorubicin was dose-related (p = 0.000). Radial strain rate and circumferential strain rate remained stable after exposure, regardless of the doxorubicin doses (Table 1, Figure 1).

### Myocardial pathology

**Figure 2:**
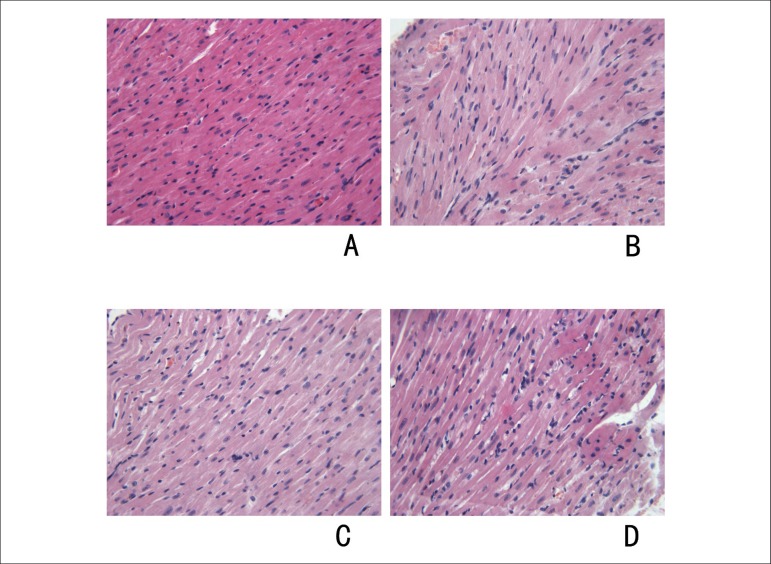
Myocardial changes after doxorubicin treatment at the light microscopy level (× 400). Vacuolization of the cytoplasm, loss of myofbrils was more severe in rats from group 4 (D). A: rat from group 1; B: rat from group 2; C: rat from group 3; D: rat from group 4.

Animals treated with doxorubicin developed cardiac lesions that could be identified on light microscopy evaluation. The characteristics of these lesions, cytoplasmic vacuolization and myofibrillar loss, have been previously observed in animal models, as well as in human patients who received anthracycline chemotherapy.^13,14^ Data on the incidence and severity of the myocardial lesions were summarized in Table 2. The severity of these lesions was significantly higher in group 3 and 4 (who received 14 mg/kg and 16 mg/kg doxorubicin, respectively) than those who received either 12 mg/kg or 10 mg/kg doxorubicin. The hearts of all animals from the control group were normal (Figure 2).

**Table 2:** Cardiomyopathy scores in Wistar rats treated with doxorubicin for 4 weeks

Dose of doxorubicin (mg/kg/w)	No. of animals	Cardiomyopathy score					
		0	1	1.5	2	2.5	3
4*	8	0	2	3	3	0	0
3.5†	7	2	2	2	1	0	0
3	8	4	2	2	0	0	0
2.5	8	6	2	0	0	0	0
Saline control	5	5	0	0	0	0	0

Cardiomyopathy scores are based on the percentage of myocytes showing cytoplasmic vacuolization and/or myofibrillar loss and are graded from 0 to 3 as follows: 0 = no alterations, 1 ≤ 5%, 1.5 = 5% to 15%, 2.0 = 16% to 25%, 2.5 = 26% to 35%, and 3 ≥ 35%.

* Cardiomyopathy scores were significantly (p < 0.05) higher than in those receiving 3 mg/kg/w or less doxorubicin.

† Cardiomyopathy scores were significantly (p < 0.05) higher than in those receiving 2.5 mg/kg/w or less doxorubicin.

### Levels of cTnI

**Figure 3:**
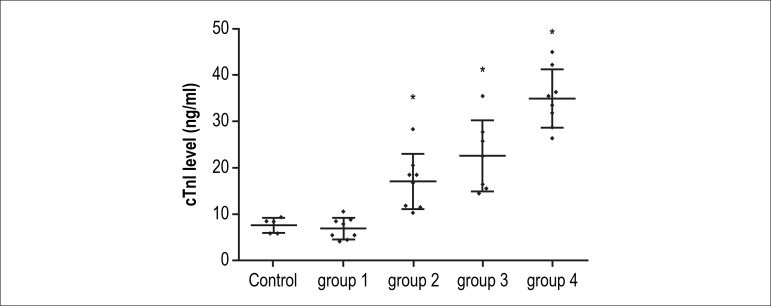
Scatter diagram of serum levels of cTnI in individual rat. *: p < 0.05 compared with that of baseline.

The levels of cTnI in the control group and groups 1,2,3, and 4 were 7.62 (3.06) ng/mL, 6.92 (4.04) ng/mL, 17.03 (8.46) ng/mL, 22.57 (12.21) ng/mL and 34.93 (11.24) ng/mL, respectively. As shown in Figure 3, the serum cTnI levels in group 1 were not significantly different from those of the control group. However, compared with those of the animals that received saline, cTnI levels significantly increased with the rise of total cumulative doses of doxorubicin (Figure 3).

### Correlations between strain values, cTnI levels and histological lesions

**Figure 4:**
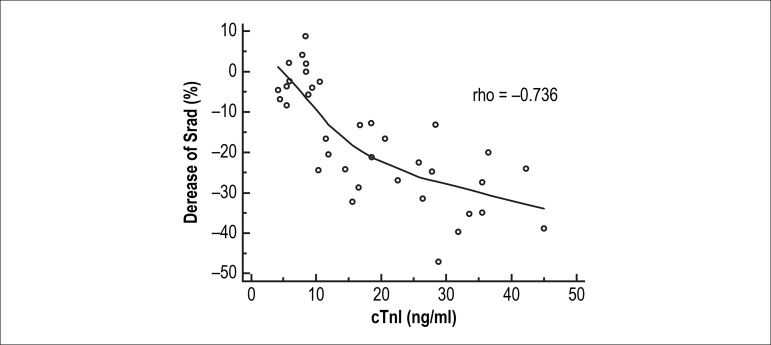
Correlations between radial strains and cTnI levels.

**Figure 5:**
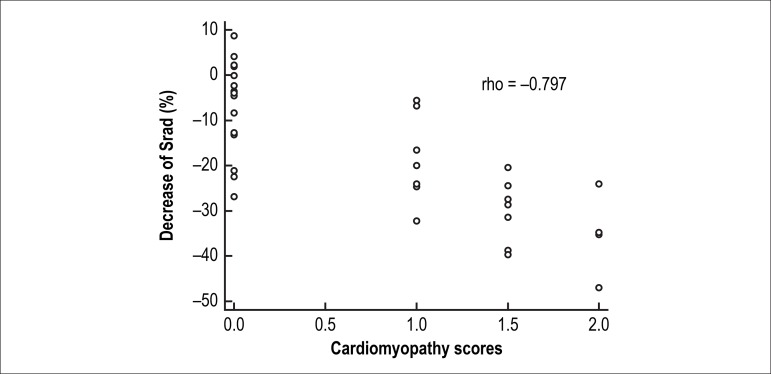
Correlations between radial strains and cardiomyopathy scores.

The decrease in radial strains exhibited a clear correlation with the cTnI levels (Spearman’s correlation rho = -0.736, p = 0.000) (Figure 4) and with cardiomyopathy scores (Spearman’s correlation rho = -0.797, p = 0.000) (Figure 5).

## Discussion

Anthracycline remains a commonly used chemotherapy agent. However, the clinical efficacy is undermined by potential life-threatening cardiotoxicity.^1^ An accurate and noninvasive method for early monitoring of cardiac injury is of vital importance to guide preventive and therapeutic strategies in reducing cardiotoxicity. In this study, we proposed a novel use of speckle-tracking imaging for the assessment of subclinical myocardial injury after anthracycline treatment. In clinical practice, LVEF monitoring is the most important clinical diagnostic tool in the recognition of cardiac dysfunction.^15,16^ However, they are somewhat insensitive in detecting early signs of cardiac stress, myocardial injury, and changes in myocardial compliance. In the present study, even though myocardial lesions and elevation of serum cTnI levels were observed after the treatment, LVEF remained stable and within the normal range, showing that LVEF was insensitive in early detection of myocardial injury.

Strain is a dimensionless parameter representing the deformation of a myocardial segment in relation to its original dimensions within a systolic time-frame. Early studies reported that the reduction in left ventricular function, caused by anthracycline, could be assessed by strain and strain rate indices when measured by Doppler imaging.^17^ With the advantages of angle independence, speckle-tracking imaging, a relatively new and more comprehensive technique, could assess both regional and global left ventricular myocardial deformation in three dimensions, providing reliable and sensitive parameters for early cardiac injury detection. Shi et al.^18^ found that radial strain analysis based on STI could detect acute allograft rejection in a rat heart transplant model, which was more sensitive than conventional echocardiographic parameters. In a rat model of athlete’s heart, speckle-tracking based strain values correlated well with pressure-volume loop-deprived contractility indices.^8^ Previous studies observed that strains decreased significantly after epirubicin treatment, although conventional echocardiographic parameters remained stable and within normal range.^19^

We demonstrated that in our animal model, radial strain was more sensitive than left ventricular ejection fraction in the assessment of cardiac injury at an early stage, which was confirmed by histological examination and serum cTnI. Chemotherapy-induced cardiotoxicity has a regional pattern,^20,21^ which could explain the increased sensitivity of strain values compared with the LVEF in the detection of early cardiotoxicity. In addition, we found that the changes in radial strain exhibited a clear correlation with histological lesions and elevation of cTnI levels, indicating that radial strain could accurately evaluate cardiotoxicity severity.

STI has a better spatial resolution in comparison with the tissue-Doppler imaging-based technique.^22^ In clinical use, frame rates > 90 frames/s often lead to poor speckle-tracking.^23^ However, because of their faster heart rates, higher frame rates are necessary in rodents. Transducer frequency, sector width and depth, as well as the number of crystals within the transducer will have an impact on scan line resolution, which will in turn affect the quality of speckle-tracking. With high crystal density over a very small sector width and depth, our 11.5 MHz transducer can obtain good images at high frame rates, with no loss of scan line resolution.

### Limitations

Cardiac imaging of the left ventricle in rodents is limited to a few echocardiographic views. Although it is possible to obtain an apical 4-chamber view, the lateral wall is rarely visualized.^24,25^ The image quality of the longitudinal view was poor and, therefore, we could not provide data about the longitudinal function.

The type of anesthesia can influence heart rate and intrinsic myocardial contractility. However, in this study, all animal including the treated and control groups underwent the same anesthesia procedure in order to limit the effect of anesthesia on cardiac function analysis. The frame rate related to the heart cycle duration used in this study was lower than studies performed in humans or large animals. Of note, we had similar frame rates as in recent experiments in a rat model of myocardial infarction and acute rejection.^18^

## Conclusion

Radial strain based on speckle-tracking imaging can provide a sensitive and noninvasive strategy in early detection of doxorubicin-induced myocardial injury.

## References

[1] Barry E, Alvarez JA, Scully RE, Miller TL, Lipshultz SE (2007). Anthracycline-induced cardiotoxicity: course, pathophysiology,
prevention and management. Expert Opin Pharmacother.

[2] Amundsen BH, Helle-Valle T, Edvardsen T, Torp H, Crosby J, Lyseggen E (2006). Noninvasive myocardial strain measurement by speckle tracking
echocardiography: validation against sonomicrometry and tagged magnetic
resonance imaging.

[3] Cho GY, Chan J, Leano R, Strudwick M, Marwick TH (2006). Comparison of two-dimensional speckle and tissue velocity based
strain and validation with harmonic phase magnetic resonance
imaging. Am J Cardiol.

[4] Helle-Valle T, Crosby J, Edvardsen T, Lyseggen E, Amundsen BH, Smith HJ (2005). New noninvasive method for assessment of left ventricular
rotation: speckle tracking echocardiography.

[5] Reisner SA, Lysyansky P, Agmon Y, Mutlak D, Lessick J, Friedman Z (2004). Global longitudinal strain: a novel index of left ventricular
systolic function.

[6] Bachner-Hinenzon N, Shlomo L, Khamis H, Ertracht O, Vered Z, Malka A (2016). Detection of small subendocardial infarction using speckle
tracking echocardiography in a rat model. Echocardiography.

[7] Bachner-Hinenzon N, Ertracht O, Malka A, Leitman M, Vered Z, Binah O (2012). Layer-specific strain analysis: investigation of regional
deformations in a rat model of acute versus chronic myocardial
infarction. Am J Physiol Heart Circ Physiol.

[8] Kovács A, Oláh A, Lux Á, Mátyás C, Németh BT, Kellermayer D (2015). Strain and strain rate by speckle-tracking echocardiography
correlate with pressure-volume loop-derived contractility indices in a rat
model of athlete's heart. Am J Physiol Heart Circ Physiol.

[9] Mor M, Mulla W, Elyagon S, Gabay H, Dror S, Etzion Y (2014). Speckle-tracking echocardiography elucidates the effect of pacing
site on left ventricular synchronization in the normal and infarcted rat
myocardium. PLoS One.

[10] Koshizuka R, Ishizu T, Kameda Y, Kawamura R, Seo Y, Aonuma K (2013). Longitudinal Strain Impairment as a Marker of the Progression of
Heart Failure with Preserved Ejection Fraction in a Rat
Model. J Am Soc Echocardiogr.

[11] Martin RA, Daly A, DiFonzo CJ, de la Iglesia FA (1986). Randomization of animals by computer program for toxicity
studies. J Environ Pathol Toxicol Oncol.

[12] Billingham ME, Silver MD (1991). Role of endomyocardial biopsy in diagnosis and treatment of heart
disease. Cardiovascular pathology.

[13] Ferrans VJ, Sanchez JA, Herman EH, Muggia FM, Green MD, Speyer JL (1992). Pathologic anatomy of animal models of anthracycline-induced
cardiotoxicity. Cancer treatment and the heart.

[14] Ferrans VJ, Sanchez JA, Herman EH, Muggia FM, Green MD, Speyer JL (1992). Role of myocardial biopsy in the diagnosis of anthracycline
toxicity. Cancer treatment and the heart.

[15] Villani F, Meazza R, Materazzo C (2006). Non-invasive monitoring of cardiac hemodynamic parameters in
doxorubicin-treated patients: comparison with
echocardiography.

[16] Walker J, Bhullar N, Fallah-Rad N, Lytwyn M, Golian M, Fang T (2010). Role of three-dimensional echocardiography in breast cancer:
comparison with two-dimensional echocardiography, multiple-gated acquisition
scans, and cardiac magnetic resonance imaging.

[17] Piegari E, Di Salvo G, Castaldi B, Vitelli MR, Rodolico G, Golino P (2008). Myocardial strain analysis in a doxorubicin-induced
cardiomyopathy model.

[18] Shi J, Pan C, Shu X, Sun M, Yang Z, Zhu S (2011). The role of speckle tracking imaging in the noninvasive detection
of acute rejection after heterotopic cardiac transplantation in
rats.

[19] Kang Y, Cheng L, Li L, Chen H, Sun M, Wei Z (2013). Early detection of anthracycline-induced cardiotoxicity using
two-dimensional speckle tracking echocardiography. Cardiol J.

[20] Ho E, Brown A, Barrett P, Morgan RB, King G, Kennedy MJ (2010). Subclinical anthracycline- and trastuzumab-induced cardiotoxicity
in the long-term follow-up of asymptomatic breast cancer survivors: a
speckle tracking echocardiographic study. Heart.

[21] Perel RD, Slaughter RE, Strugnell WE (2006). Subendocardial late gadolinium enhancement in two patients with
anthracycline cardiotoxicity following treatment for Ewing’s
sarcoma.

[22] Dandel M, Hetzer R (2009). Echocardiographic strain and strain rate imaging--clinical
applications. Int J Cardiol.

[23] Suffoletto MS, Dohi K, Cannesson M, Saba S, Gorcsan J (2006). Novel speckle tracking radial strain from routine black-and-white
echocardiographic images to quantify dyssynchrony and predict response to
cardiac resynchronization therapy.

[24] Hirano T, Asanuma T, Azakami R, Okuda K, Ishikura F, Beppu S (2005). Noninvasive quantification of regional ventricular function in
rats: assessment of serial change and spatial distribution using ultrasound
strain analysis. J Am Soc Echocardiogr.

[25] Neilan TG, Jassal DS, Perez-Sanz TM, Raher MJ, Pradhan AD, Buys ES (2006). Tissue Doppler imaging predicts left ventricular dysfunction and
mortality in a murine model of cardiac injury. Eur Heart J.

